# Interferometric Deflection Analysis of Suspended 2D Polyaramid Thin Films

**DOI:** 10.1002/smtd.202501543

**Published:** 2025-12-05

**Authors:** Michelle Quien, Cody L. Ritt, Sanjay S. Garimella, Hagen Gress, Kamil L. Ekinci, Joseph Scott Bunch, Michael S. Strano

**Affiliations:** ^1^ Department of Chemical Engineering Massachusetts Institute of Technology Cambridge MA 02139 USA; ^2^ Department of Mechanical Engineering Boston University Boston MA 02215 USA

**Keywords:** 2D materials, gas barrier, interferometry, nanofilm

## Abstract

The 2D nanofilm bulge test, which uses an Atomic Force Microscope (AFM) to measure the deflection of a suspended film under various conditions, has emerged as an important measurement platform for understanding mechanical, barrier, and permeability properties of 2D materials as thickness approaches the angstrom scale. The problem considered in this work is the limitation of such bulge analyses imposed by the AFM whereby dynamic measurements under high pressure, high temperature, and chemically corrosive conditions are limited. In this work, a technique is developed for measuring nanofilm deflection using only visible light interferometry. Both theoretical and semi‐empirical models are applied to translate multicolor interference patterns from broadband excitation into estimates of nano‐film deflection, allowing nanoscale precision in most cases. The technique and algorithm advanced in this work allows the use of widespread optical microscopy to widen the study of these important 2D nanofilm systems to more relevant conditions.

## Graphical abstract

A visible light interferometry technique is developed to measure the deflection of 2D nanofilms, replacing AFM‐based methods. Using theoretical and semi‐empirical models, multicolor interference patterns from broadband excitation are translated into nanoscale deflection estimates. This approach enables studying 2D nanofilms under high pressure, high temperature, and chemically corrosive conditions using standard optical microscopy.

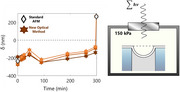

## 1 Introduction

2D nanomaterials, such as graphene, transition metal dichalcogenides, and 2D polymers, have compelling elastic,^[^ ^1^, ^2^, ^3^, ^4^, ^5^ ^]^ inelastic,^[^ ^1^, ^6^, ^7^, ^8^ ^]^ interfacial,^[^ ^9^, ^10^ ^]^ resonance,^[^ ^11^, ^12^, ^13^, ^14^, ^15^ ^]^ and gas transport and barrier^[^ ^16^, ^17^, ^18^ ^]^ properties. The bulge test platform for 2D materials, wherein the nanofilm is suspended over an etched Si/SiO_2_ well and probed with atomic force microscopy (AFM), has been pivotal in observing these properties^[^ ^1^, ^2^, ^3^, ^5^, ^7^, ^9^, ^10^, ^17^, ^18^, ^19^, ^20^, ^21^, ^22^ ^]^ as it offers access to these properties at the scale of nanometer thickness. Moreover, these tests allow for extremely sensitive measurements, such as the ultra‐low impermeability of graphene or single nanopore gas permeation,^[^ ^17^, ^21^, ^23^ ^]^ Such measurements are not possible in equivalent macroscale experiments. The problem considered in this work is the limitation of such bulge analyses imposed by the use of the Atomic Force Microscope (AFM) as the sensitivity readout of the membrane defection. While sensitive to as low as 100 pm change in deflection, standard AFMs require highly precise control over the cantilever and thus are best suited for low temperatures,^[^ ^24^ ^]^ low energy, and low pressure, inert gas conditions.^[^ ^25^ ^]^ Advancements in AFM have supported imaging in liquid and biological media, but has yet to extend to high temperature, pressure, or chemically corrosive conditions.^[^ ^26^ ^]^ This limits the study of 2D nanofilms in many important technological areas. In this work, we develop a technique for measuring nanofilm deflection using only visible light interferometry, allowing the use of widespread optical microscopy to widen the study these important systems. We apply both theoretical and semi‐empirical models to translate multicolor interference patterns from broadband excitation into estimates of nanofilm deflection, allowing nanoscale precision in most cases.

As relatively new nanotechnology systems, 2D polymers have attracted recent attention^[^ ^27^, ^28^, ^29^, ^30^ ^]^ as a means of combining the mechanical strength and in‐plane energy conduction of conventional 2D materials^[^ ^1^, ^31^, ^32^, ^33^ ^]^ with the low densities, synthetic processability, and organic composition of their 1D counterparts. Recent theoretical^[^ ^34^ ^]^ and experimental^[^ ^35^ ^]^ work by our group has enabled the irreversible, solution‐phase polymerization of 2D polyaramids. Solution processing at preparative scales of these 2D polyaramids have already achieved highly oriented, large area free‐standing films which exhibit exceptional 2D elastic modulus and yield strength already reaching 12.7 and 0.5 GPa, respectively.^[^ ^35^ ^]^ These ultra‐thin, high mechanical strength films have the potential to excel as gas membranes and/or barrier materials.^[^ ^36^ ^]^

Historically, thin film optical interference has been a powerful and versatile tool for probing the properties of 2D nanomaterials. Critically, the isolation of single‐layer graphene was possible because of its layer‐dependent light interference pattern with SiO_2_ wafers.^[^ ^37^, ^38^ ^]^ The reflected light from a thin film system is indicative of the number of 2D nanomaterial layers,^[^ ^39^, ^40^, ^41^, ^42^ ^]^ electronic band structures,^[^ ^43^ ^]^ and even defects^[^ ^44^, ^45^ ^]^ in the film. This tool thus enables a precise, non‐destructive analysis of nanomaterial structural, electronic, and optical properties, critical for both fundamental research and device development. Furthermore, we pursue the use of a broadband light spectrum to make this tool as accessible as possible.

Herein, we develop a method to measure 2D nanofilm bulge deflection using broadband excitation and visible light interference patterns. This allows the use of common optical microscopes to perform sensitive 2D nanofilm deflection measurements under a much wider range of conditions than previous possible. We apply both theoretical and semi‐empirical models to such complex interference patterns, demonstrating greater success with the latter. The approach creates a calibration curve to semi‐empirically correlate red (R), green (G), and blue (B) pixel values with AFM height measurements. Our work develops a MATLAB algorithm that converts the visible, optical interference pattern into an estimate of bulge deflection. We evaluate several pressurization experiments at 150 kPa on 2D polyaramid samples within an microscopy environmental chamber to demonstrate the technique.

## 2 Converting Visible Light Interference to Nanofilm Deflection

### 2.1 Pressurization Experiment on 2DPA‐1

**Figure 1:**
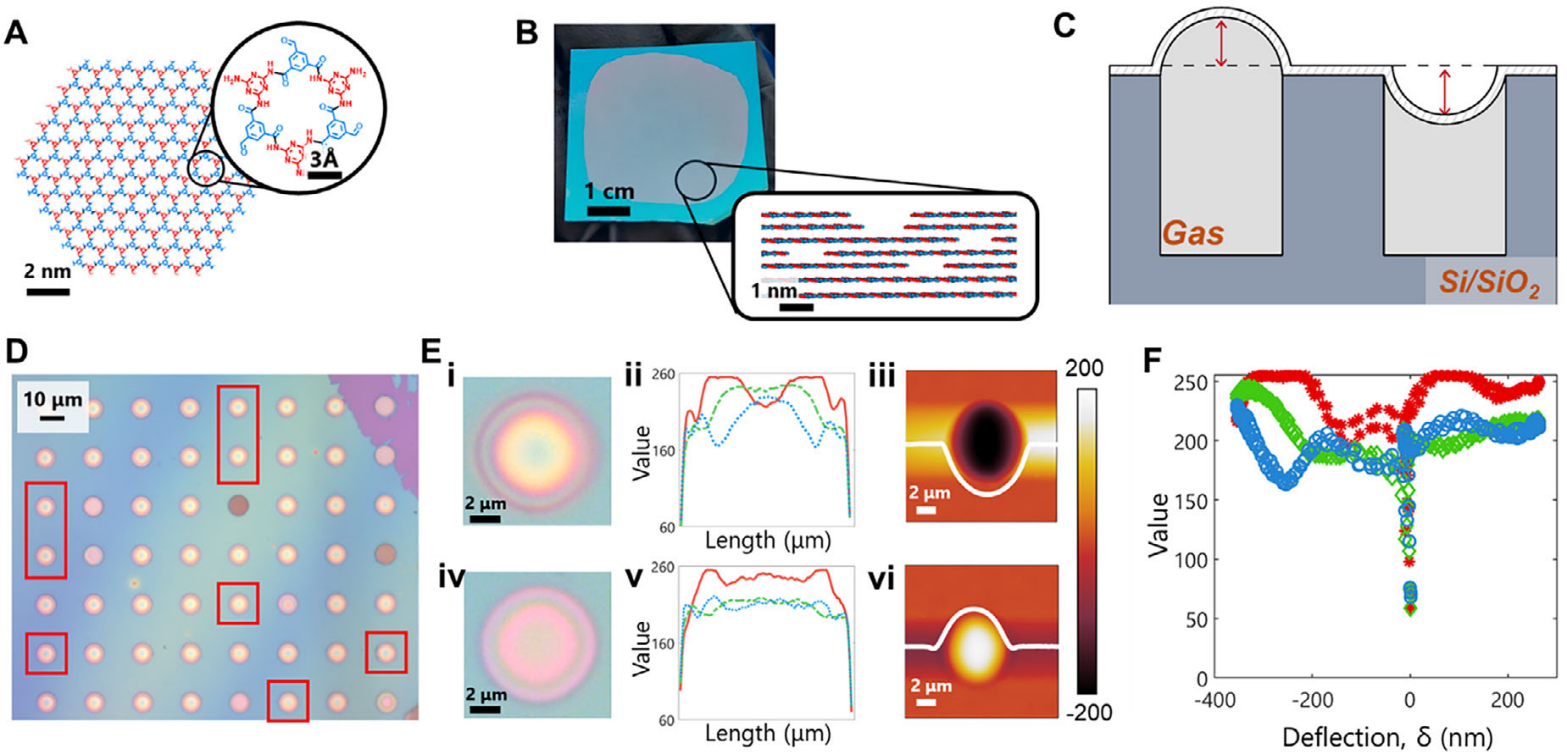
Problem Description for 2D Nanofilm Deflection Measurement, A) Molecular structure of a single hexagonal 2DPA‐1 platelet. Inset: chemical structure of a single macrocycle. B) Spin‐coated thin film of 2DPA‐1 on a Si/SiO_2_ substrate. Inset: stacking configuration of 2DPA‐1 platelets within the thin film. C) Schematic of two possible deflections within the bulge test set‐up. A 2D nanofilm (white) is suspended over microwells filled with gas of interest (light gray) that are patterned into a Si/SiO_2_ substrate (dark gray). Deflections are measured from the top of the film (red arrows). D) Optical micrograph of a bulge test sample. Bulges monitored for this study (red squares) were initially downward deflected. E) Correlation between an optical micrograph of a bulge (i, iv), the trend in RGB pixel values across the bulge (ii, v), and the measured AFM height profile (iii, vi). Shown for both an upward deflected (top row) and downward deflected (bottom row) bulge. R component of RGB is represented with a red solid line, G represented with a green dashed line, and B represented with a blue dotted line. F) Correlation between RGB pixel values and the corresponding AFM measured deflection for both the upward and downward deflected bulges shown in E. Red asterisks correspond to the R component of RGB, green diamonds correspond to G, and blue open circles correspond to B.

2D polymers as a class of materials are intriguing for their gas barrier and membrane applications because of their controllable pore size and pattern and enhanced processability. 2DPA‐1, or 2D polyaramid, form platelets up to 10 nm in diameter with each macrocycle unit ≈9Å in diameter (**Figure** **1**A). Uniquely processable for a 2D polymer, this material can be dissolved in trifluoroacetic acid (TFA) and spin‐coated into nanofilms as wide as 6 inches in diameter, and based on their undetectable BET surface area in N_2_ and DFT simulations,^[^ ^35^ ^]^ are hypothesized to form an overlapping platelet structure (Figure 1B). Nanofilm bulge test pressurization experiments are thus crucial to determining the lower limit of 2DPA‐1′s permeability.

In the bulge‐test pressurization experiment, nanofilms are suspended over wells etched into an Si/SiO_2_ wafer (Figure 1C). The deflection of the nanofilm, δ, is indicative of the gas pressure within the wells and is describable with Hencky's solution.^[^ ^46^ ^]^ Thus, as gas permeates through the nanofilm, the deflection of the film decreases, and the permeability can be obtained.

To prepare a sample for the bulge‐test pressurization experiments, we transfer 2DPA‐1 spin‐coated films, as we have produced elsewhere,^[^ ^35^ ^]^ 35 nm thick, over wells patterned into an Si/SiO_2_ wafer (1‐um deep, 8.5 µm wide) (Figure 1D), and for this analysis we monitored 8 bulges. We controllably create negatively‐deflected bulges by annealing our samples above atmospheric temperature at 50 C (Figure 1E‐i). We optically measure the R, G, and B pixel values across the bulge (Figure 1E‐ii) as well measure with AFM how δ varies across the bulge (Figure 1E‐iii).

In the pressurization experiment, we used an environmental chamber to subject the sample to 150 kPa of N2 above atmospheric for 5 h, obtaining 9 camera images of the bulges throughout the process through an optical microscope. At the end of the pressurization, all 8 monitored bulges show a new R, G, and B value trend across the bulge (Figure 1E‐iv,v). Subsequent AFM measurements show that the bulges all became positively deflected (Figure 1E‐vi), thus showing they were filled with N2 throughout the experiment. Altogether, there is a distinct trend between R, G, and B pixel values with both positive and negative δ (Figure 1F). We note that the discontinuity ≈0 nm corresponds to edge effects and the adhesion of the film to the etched well walls, and thus will not be including the data points from −100 to 15 nm for this study.

### 2.2 Theoretical Modeling of Visible Interference in the Nanofilm

**Figure 2:**
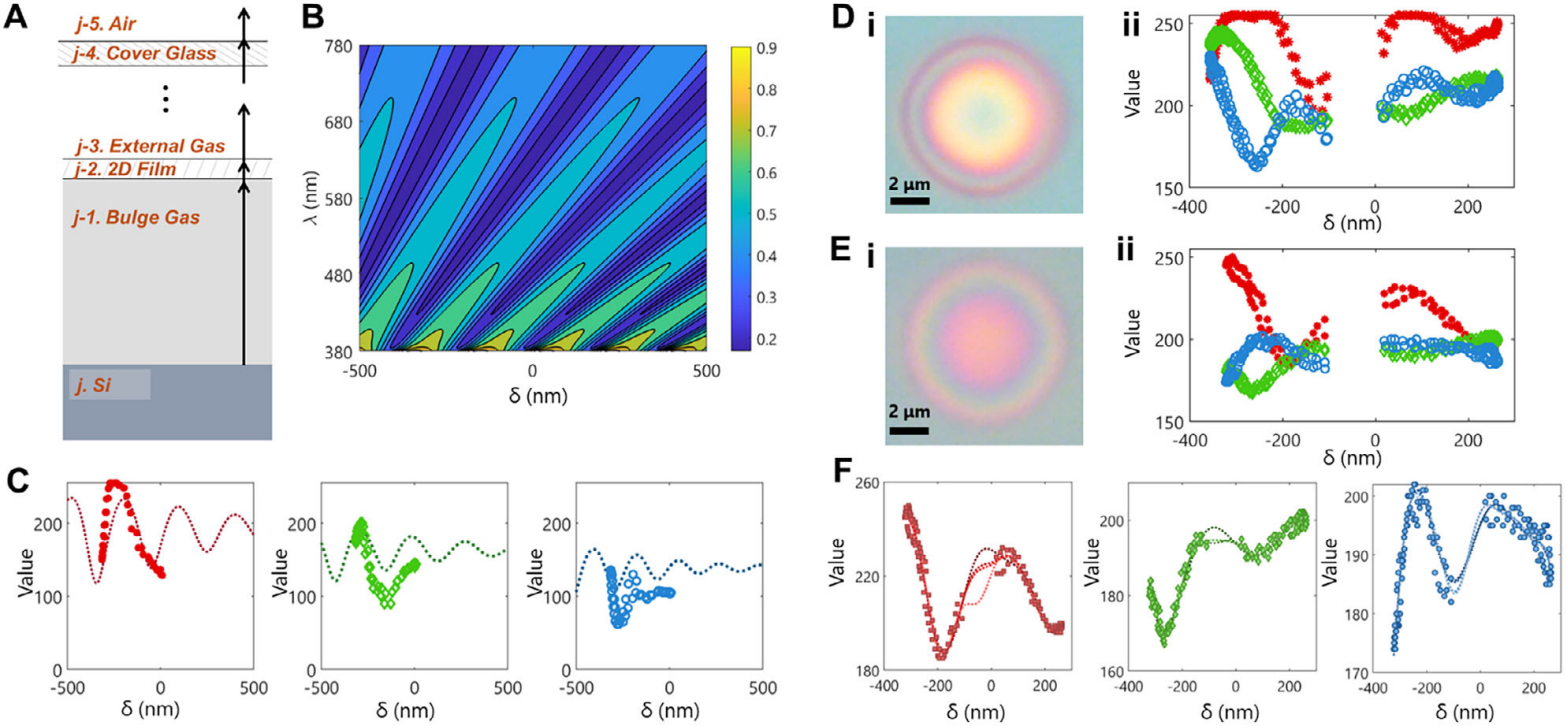
Theoretical and Semi‐Empirical Model for Converting Visible Light Interference to Observed Predicted RGB Values. A) Schematic of normal light reflectivity through 3 interfaces present in the experimental set‐up. B) Contour plot of the reflected normal light intensity (range 0 to 1) for the full range of experimentally measured film depths (‐500 to 500 nm) and the full range of visible wavelengths (380 to 780 nm). C) Comparison of calculated RGB pixel values for various film depths (dotted lines) to experimentally measured RGB pixel values (markers). Shown for each RGB channel (left, R; center, G; right, B). D) Optical micrograph of a bulge without a glass cover (i) and the correlation between the RGB pixel values across the bulge with the measured AFM profile (ii). E) Optical micrograph of a bulge under a glass cover (i) and the correlation between the RGB pixel values across the bulge with the measured AFM height profile (ii). F) Comparison of fit Fourier sum equations to experimentally measured RGB pixel values from the glass‐covered sample (markers). Shown for each RGB channel (left, R; center, G; right, B). Each dashed line represents a different Fourier fit (3‐term, 4‐term, and 5‐term, darkest to lightest respectively).

The Fresnel equations can describe the intensity of light reflected at an interface or a series of interfaces. In our simplest case, where the sample is not contained in an environmental chamber, there are 3 interfaces, j, relevant for light reflection (**Figure** **2**A and j = 3). For this case, the intensity of light reflected normal to the sample, *I*, is described with Equation 1:^[^ ^39^ ^]^

(1) $$I\left(\lambda \right)={\left|\frac{r_{1}e^{i\phi_{a}}+r_{2}e^{-i\phi_{b}}+r_{3}e^{-i\phi_{a}}+r_{1}r_{2}r_{3}e^{i\phi_{b}}}{e^{i\phi_{a}}+r_{1}r_{2}e^{-i\phi_{b}}+r_{1}r_{3}e^{-i\phi_{a}}+r_{2}r_{3}e^{i\phi_{b}}}\right|}^{2}$$

where

(2) $$r_{1}=\frac{n_{0}-n_{1}}{n_{0}+n_{1}}$$

(3) $$r_{2}=\frac{n_{1}-n_{2}}{n_{1}+n_{2}}$$

(4) $$r_{3}=\frac{n_{2}-n_{3}}{n_{2}+n_{3}}$$

(5) $$\phi_{1}=\frac{2\pi n_{1}d_{1}}{\lambda }$$

(6) $$\phi_{2}=\frac{2\pi n_{2}d_{2}}{\lambda }$$

(7) $$\phi_{a}=\phi_{1}+\phi_{2}$$

(8) $$\phi_{b}=\phi_{1}-\phi_{2}$$

Here, *n*_0_, *n*_1_, *n*_2_, and *n*_3_ are the complex refractive indices of layers 0, 1, 2, and 3, respectively, and *d*_1_ and *d*_2_ are the thicknesses of layers 1 and 2, respectively. Layers 0 and 2 are gas and thus *n*_0_ = *n*_2_ = 1. Layer 3, silicon, has a known wavelength‐dependent complex refractive index.^[^ ^47^ ^]^ We measured *n*_1_, the wavelength‐dependent complex refractive index of 2DPA‐1, using a Filmetrics F20 reflectometer (Figure S1, Supporting Information). *d*_2_ is related to δ via the well depth: δ = *d*_2_ − 1µ*m*. Thus, Equation 1 simplifies to be dependent on only two variables: *I* = *f*(δ, λ). We then can calculate the reflected normal intensity, ranging from 0 to 1, of each visible wavelength for a different δ of a 2DPA‐1 thin film (Figure 2B).

To convert reflected light intensity to the pixel values in an optical microscopy image, we account for the spectral power density of our light source and assume the camera's wavelength sensitivity such that it is a standard observer in the CIE XYZ color system. We compare the computed RGB values for different film depths with experimental results for a negatively‐deflected bulge in Figure 2C. We find that both the computed and experimental trends have similar sinusoidal decay nature.

However, we find that the theoretical approach has practical limitations. Regarding the Fresnel equations, there is the assumption that the light detected is reflected normal (which is not true for the entire field of view of the sample) at each interface (which the lithographically etched wells do not have nanometer‐precise vertical walls). Moreover, the conversion from detected light intensity to RGB values would be more thorough if we measured our light source's specific spectral power density with a spectroradiometer and if we had generated the color matching functions and transformation matrices for our specific camera, which is a non‐trivial task. To do the latter, there is a color‐based method which involves using color samples and imaging them individually with a spectrophotometer and digital camera, ideally for each wavelength of interest, and then generating transformation matrices to convert detected intensities to RGB values.^[^ ^48^ ^]^ These discrepancies, specifically for the conversion of detected light intensity to RGB values, account for the differences between theoretical and experimental RGB values in Figure 2C.

### 2.3 A Semi‐Empirical Description of Visible Light Interference

In addition to the aforementioned assumptions, in our experiment, we note that the pressure chamber has a glass cover. This would add two extra interfaces at which light can be reflected (Figure 2A and j = 5). Experimentally, we observe a difference between the RGB trends of the sample when there is no glass cover (Figure 2D‐i,ii) and when there is a glass cover (Figure 2E‐i,ii). The glass‐covered system is theoretically described by a variation of Equation 1 that has 16 terms in the numerator and 16 terms in the denominator.^[^ ^39^ ^]^ Our approach is to use a Fourier series to semi‐empirically describe the experimental data (Figure 2F, Equation 9) in a tractable form. Given that we removed the data points from −100 to 15 nm because of edge effects, we varied the number of terms in the Fourier series fits to account for a range of possible fits in the region. We used MATLAB's Curve Fitting Toolbox for each fit, and used the 3‐term, 4‐term, and 5‐term options, which uses the functional form:

(9) $$RGB=\sum_{n=0}^{N}a_{n}\cos\left(n*w*\delta \right)+b_{n}\sin\left(n*w*\delta \right)$$

where *RGB* is the pixel value for the R, G, and B channels of the image, *N* is the number of terms in the fit (in this case, 3, 4, and 5), and *a_n_*, *b_n_*, and *w* are terms fit by the MATLAB toolbox. We performed a fitting for R, G, and B values with AFM height separately.

### 2.4 Processing Algorithm

With the fit R, G, and B equations established for positive and negative deflections, we coded an algorithm in MATLAB to convert the RGB data within a bulge microscopy image into a height profile. The algorithm consists of two codes: the first approaches the bulge from a 3D level, the second interprets individual 2D slices from the full 3D bulge. The first code discretizes the bulge image into diameter slices in cylindrical coordinates, which then is input individually into the second code. The second code calculates and returns the height results for individual diameter slices, which the first code reconstructs into a full 3D bulge profile.

**Figure 3:**
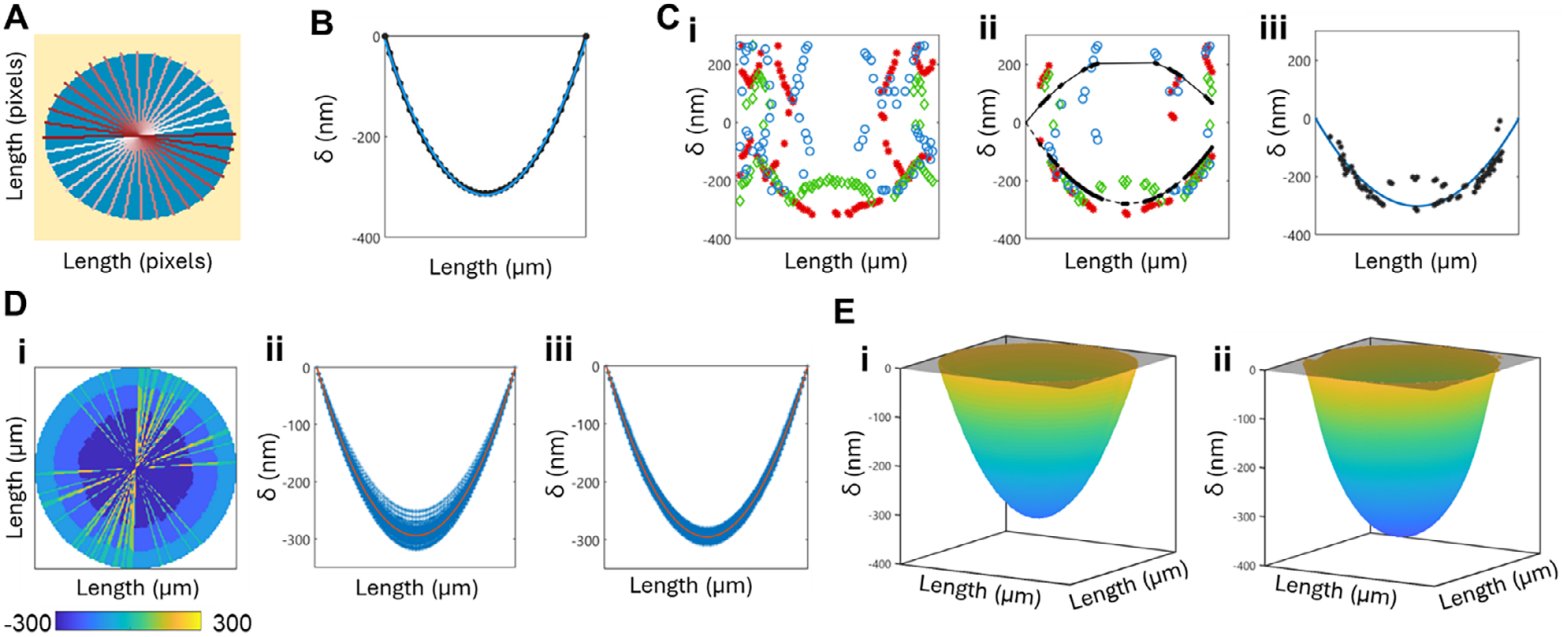
Algorithmic Processing of Visible Light Interference from Optical Micrographs to Film Deflection. A) Automated masking of a bulge optical micrograph and subsequent discretization for analysis. The bulge (blue) is distinguished from the flat background (yellow). The bulge is discretized into diameters at user‐specified ϑ spacing, shown for 10°. Diameters are light red to dark red lines, where the lightest line is the first diameter analyzed and the darkest red line is the last diameter. B) Process for calculating the height profile of a single diameter slice. (i) Heights calculated for each pixel of the diameter slice using the Fourier sum fits for R, G, and B. (ii) Remaining R,G, and B height solutions once the local slope criteria for physically possible bulge shapes is applied. Two parabolas are fit, one to the positive height solutions, one to the negative height solutions. (iii) The final parabola fit for the diameter slice. The negative or positive deflected parabola is chosen based on goodness of fit. A stepwise regression removes solutions that are extraneous. C) Comparison of a bulge profile calculated with Hencky's solution (black line, black asterisks) and a parabola constrained to 0 at the edges of the bulge (blue line) D) Process for generating a single 3D bulge profile. (i) The final parabola fit for each diameter slice is compiled into a single bulge image. The bulge is determined to be upward or downward deflected based on the majority deflection in the parabola fits. (ii) All parabolas corresponding to the determined bulge deflection are fit to a single parabola. (iii) Stepwise regression removes parabola solutions that are outliers and generates a final single parabola fit. E) Final generated circular paraboloid bulge profile (i) compared to the 3D AFM measurement of the same bulge (ii). Note that the bulge shape is physically related to Hencky's solution, which is not a circular paraboloid. Gray transparent plane indicates 0 nm.

First, we establish a mask of the bulge (**Figure** **3**A). We input an image of the bulge wherein we have removed the background film using standard background removal software. Then, we calculate the pixel length of the diameter. Pixel positions are calculated for each diameter at user‐defined ϑ values, evenly spaced from 0 to 180 degrees. The RGB values of each diameter slice are then input into the second code to be converted into a height profile.

Theoretically, the profile of a bulge is described by Hencky's solution, which assumes uniform lateral loading across the bulge's surface:^[^ ^46^, ^49^ ^]^

(10) $$\frac{\delta }{d/2}\left(r\right)={\left(\frac{\Delta p}{2E}\right)}^{\frac{1}{3}}\sum_{0}^{\infty }a_{2n}\left(1-{\left(\frac{r}{d/2}\;\right)}^{2n+2}\right)$$

where δ is deflection, r is the radial position from the center of the bulge, Δp is the pressure difference across the bulge, d is the bulge diameter, E is the elastic modulus, and a_2n_ are coefficients related to the nanofilm's Poisson's ratio, ν. We find that this functional form is well approximated by a first‐term approximation of Equation 10, or a second‐order polynomial (Figure 3B). We had calculated the discrepancies caused by this approximation compared to the two‐term, three‐term, and four‐term approximations (Figure S2, Supporting Information) and find that the area under the curve of the first‐term approximation varies by at most 3% from that of the four‐term approximation (Figure S2F, Supporting Information) for ν ∈ [0,  0.5], which is what's relevant for calculations regarding the pressure, moles of gas, etc., within the bulge.

For each diameter slice, using the 2D code, a parabolic height profile is calculated from the RGB values (Figure 3C‐i–iii). First, using the Fourier fits (Figure 2F), height values are calculated from each R, G, and B fit. The Fourier fits are not one‐to‐one functions, thus resulting in up to 4 solutions per R, G, and B fit (Figure 3C‐i). We then remove solutions that do not contribute to physically possible bulge profiles and fit a parabola to the most viable height solutions (Figure 3C‐ii). If two neighboring solutions have a physically unlikely slope, then they are removed from the data set. We also remove solutions at the edges because the edges do not follow the same R, G, and B fits due to the film adhesion to the well walls. Next, we determine whether the fit will be a positive or negative deflection. The data set is split into positive height values and negative height values, and a second‐order polynomial is fit using MATLAB's lsqcurvefit function. The fits are constrained such that the height is 0 at the end points of the diameter. Then, the R2 of each fit and the number of data points corresponding to each fit is calculated, and based on the ratio of these values between both fits, the negative or positive fit and corresponding data points are removed. Finally, a stepwise regression is performed on the remaining data points, using standard error as the criteria. The resulting parabolic fit is output from this code (Figure 3C‐iii). Of note, the user controls the criteria for the slope, ratio of R2 values, and the stepwise regression. For this study, we determined these criteria such that the calculated heights of bulges with experimental AFM data agreed with the experimental data in terms of deflection direction.

Once a height profile is calculated for each diameter slice (Figure 3D‐i), the overall deflection of the bulge is fit to create a smooth bulge profile. First, all the negative and positive height profiles are counted, and the minority is removed. The remaining height profiles are fit to a second order polynomial using *lsqcurvefit* with the criteria that the end points are 0 (Figure 3D‐ii). Then, a stepwise regression is performed, using standard error as the criteria, and data points are removed if the standard error is too large. A final parabola is fit to the remaining data points (Figure 3D‐iii). Finally, the final parabola is assumed to be the slice of a circular paraboloid, and thus the algorithm outputs a deflection profile with the functional form of a circular paraboloid (Figure 3E‐i). We show the comparison of a calculated bulge profile (Figure 3E‐i) with the corresponding AFM measurement result (Figure 3E‐ii).

## 3 Algorithmic Results and Discussion

### 3.1 Algorithmic Interpretation of Pressurization Experiments

**Figure 4:**
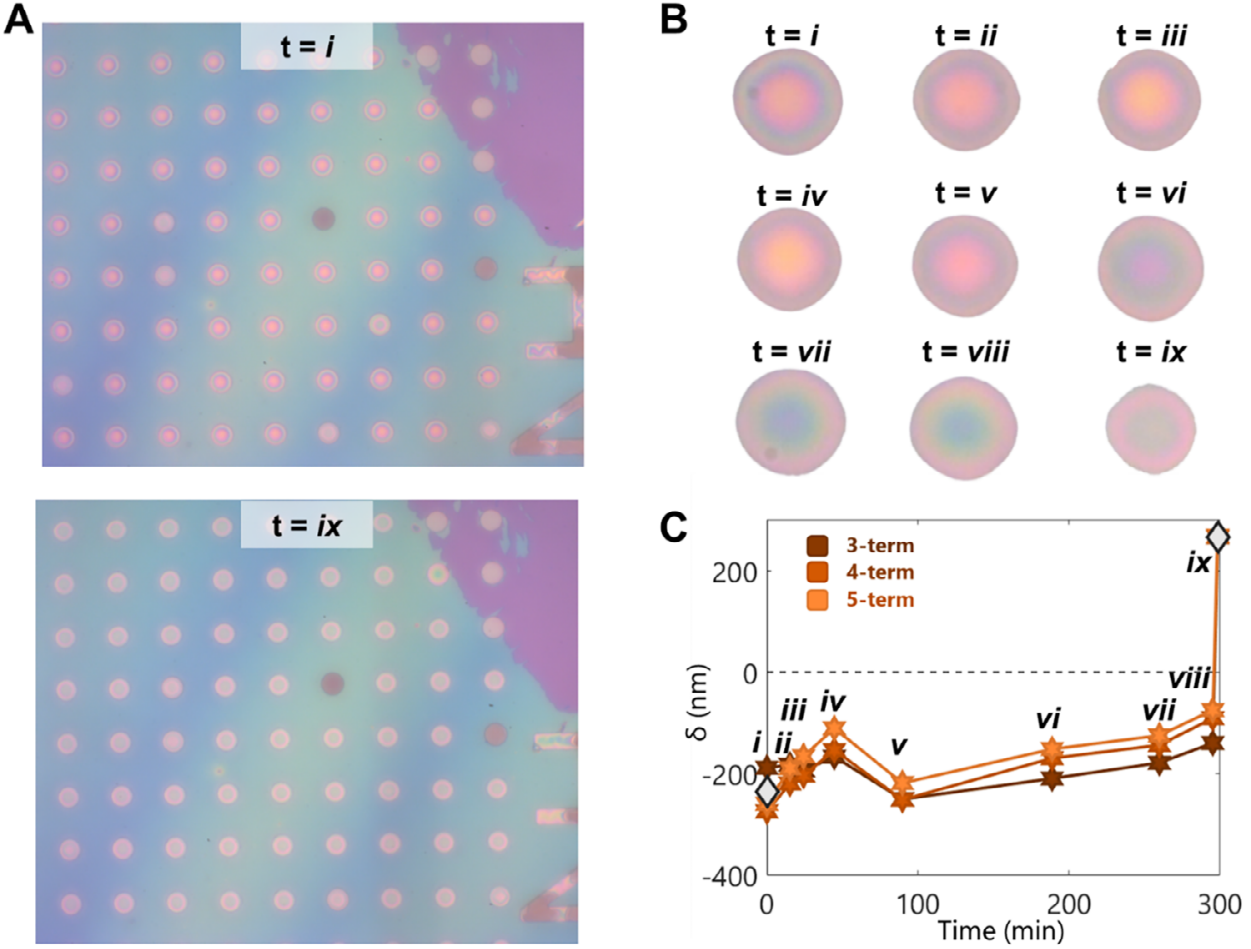
Bulge Images throughout Pressurization, A) Image of the sample before (*t = i*) and after (*t = ix*) N_2_ pressurization at 150 kPa. B) Isolated images of a single bulge, also corresponding to Figure 5B i, throughout the pressurization at all 9 time points. C) Corresponding heights calculated from the isolated bulge images.

**Figure 5:**
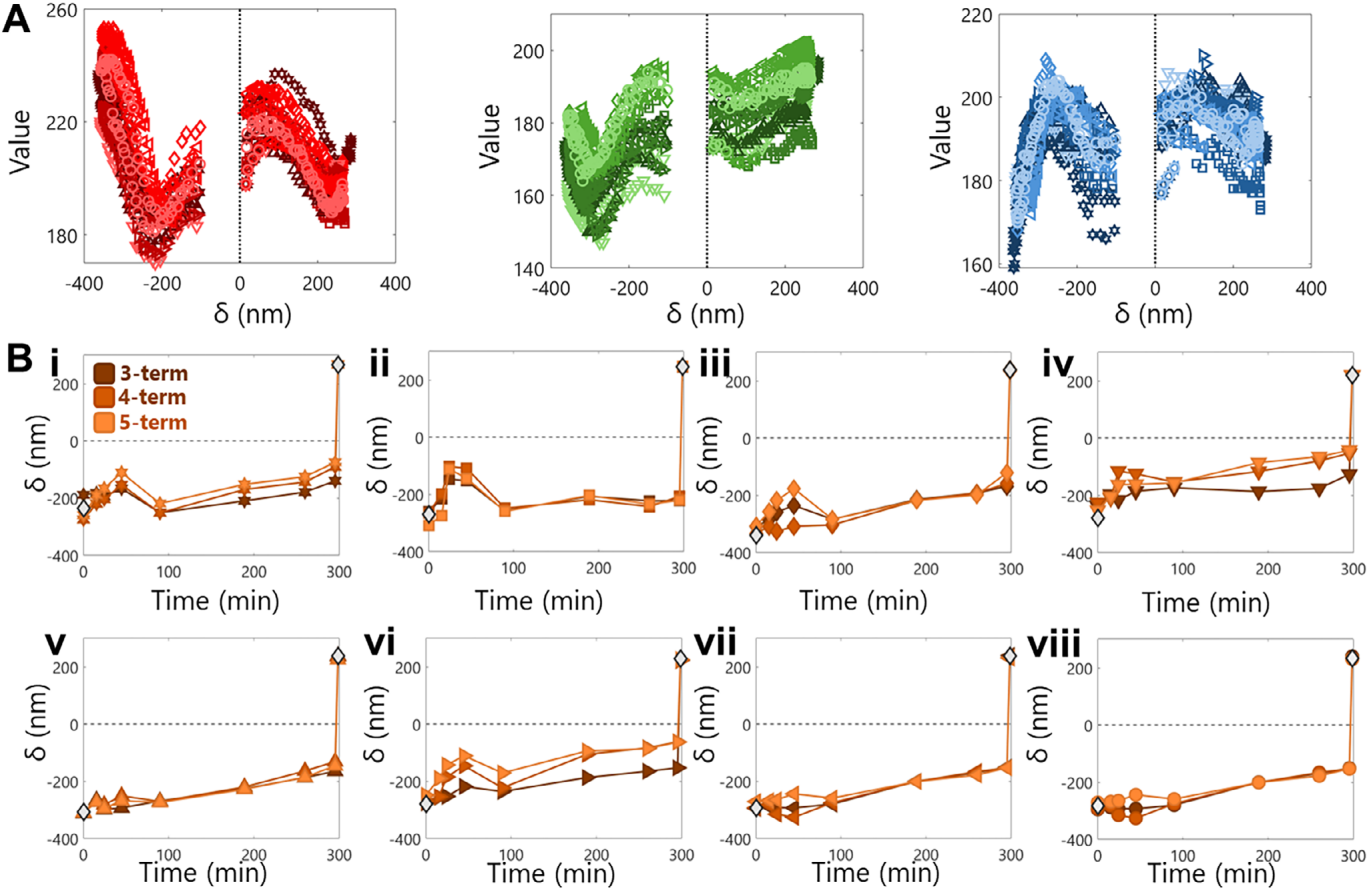
Evaluation of Self Consistency and Precision for Calculated Nanofilm Deflections. A) R (top), G (middle), and B (bottom) pixel values correlated with AFM measurements for all 8 studied bulges. A black dotted line delineates positive from negative height values. Each marker shape corresponds to one bulge. B) Calculated deflection extrema (orange markers) are plotted for one full pressurization experiment on all 8 studied bulges. Within each plot, three data sets are shown for the 3‐term, 4‐term, and 5‐term Fourier fits (darkest to lightest, respectively). The extrema of measured AFM profiles (white diamonds) are shown where obtained. A black dotted line delineates positive from negative height values. Each marker shape corresponds to one bulge.

We applied our algorithm to 9 optical microscopy images of 8 bulges (Figures 1D and **4**, Figure S3, Supporting Information) over the course of our 6‐h pressurization experiment. For each of the time points, we captured a region ≈150 by 150 µm in area (Figure 4A). Then we isolated each bulge individually from the background substrate (Figure 4B), which corresponds with each of the time points analyzed in Figures 4C and **5**.

Each of the 8 bulges have slightly different R, G, and B fit and trends (Figure 5A), which we attribute to differences in light reflection angles and potential differences in the glass cover position, as it can warp during the pressurization. We used our algorithm to calculate the full bulge profile for all images and show the trend in the extrema of the deflection throughout the experiment (Figure 5B). We also calculated these extrema for each of the Fourier fits (3‐term, 4‐term, 5‐term) (Figure S3, Supporting Information).

**Figure 6:**
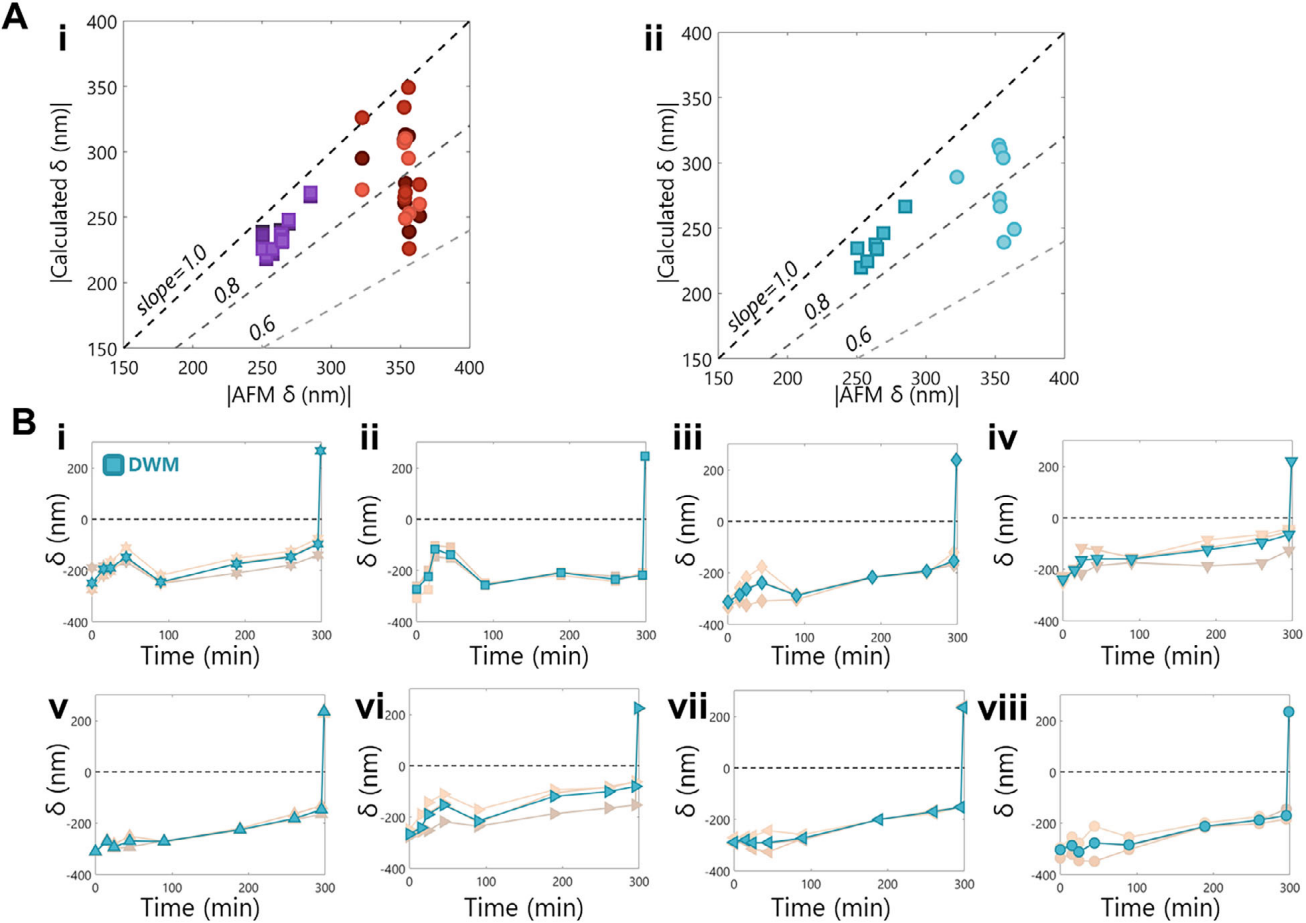
Use of DWM to Refine Precision. A) Parity plot of the absolute value of the initial and final data points for all 8 studied bulges, for each of the 3‐term, 4‐term, and 5‐term Fourier fits (i) and for the DWM (ii). Dashed lines have slopes 1.0, 0.8, and 0.6, darkest to lightest, respectively. Square data sets correspond to the positive extrema while circle data sets correspond to negative extrema. Three sets of each color are shown for the 3‐term, 4‐term, and 5‐term Fourier fits (darkest to lightest, respectively). B) Calculated DWM of the deflection extrema (blue markers) are plotted for one full pressurization experiment on all 8 studied bulges. Within each plot, we include the 3‐term, 4‐term, and 5‐term Fourier fits, at high transparency (darkest to lightest, respectively). A black dotted line delineates positive from negative height values. Each marker shape corresponds to one bulge.

We used a parity plot to quantify the accuracy of the fittings (**Figure** **6**A‐i). We find that the calculated positive deflections have better parity with the AFM data than the calculated negative deflections, regardless of the number of Fourier fitting terms. This is likely a consequence of the relationship between RGB and height not being a one‐to‐one functional mapping, as the number of possible solutions is greater for negative deflections than positive deflections (Figure 2F). We also note that all of the fits on the parity plot are less than unity. This is likely because the algorithm always accounts for interpolated solutions, and the data points in the parity plot correspond to the highest and lowest measured deflections in the entire data sets.

We wish to understand the real fluctuation of these bulges during the pressurization experiment. As we found all 3 fits showed similar parity, we introduce a quantity that we call the Distance Weighted Mean (DWM) for each time point using the 3‐term, 4‐term, and 5‐term Fourier fits (Figure 6A‐ii,B). The DWM (Equations 11 and 12) weighs the results of each of the fits based on how distant they are from each other, thus accounting more for higher agreement between the fits:

(11) $$DWM=\frac{\sum_{i=1}^{3}w_{i}\delta_{i}}{\sum_{i=1}^{3}w_{i}}$$

where

(12) $$w_{i}=\frac{1}{\sum_{j=1}^{3}\left|\delta_{i}-\delta_{j}\right|}$$

The weighting of this metric toward data point agreement is exhibited in the parity plot (Figure 6A‐ii) as there is less variation in the DWM parity values as opposed to the individual fitted results (Figure 6A‐i).

We note two distinct regimes of behavior in the time series of the DWM results: before 100 min, and post 100 min. We find that 4 of the 8 bulges increase, then decrease, in height before 100 min (Figure 6B‐i–iii,vi), while the remaining 4 bulges appear to quickly stabilize their heights before 100 min (Figure 6B‐iv,v,vii,viii). While these 8 bulges are all on the same sample, this difference in behavior is likely caused by differences in how well the rim of the bulges are adhered to the substrate. Then, after 100 min, 7 of the 8 bulges steadily increase in height for the remainder of the experiment (Figure 6B‐i,iii–viii). This could be evidence that the rim seal has been opened and all 7 of the bulges are steadily filling with pressurization gas. Moving forward, this analysis will be key to understanding the filling behavior and rim seal mechanics of 2D nanofilm bulges, as we can now probe these bulges in different environments.

### 3.2 Applying the Algorithm

We detail below how to extend this algorithm to other systems, referencing our usage of this on an experiment in which we monitor 8 bulges that are initially inflated with nitrogen, subjected to helium, and then deflated over time (Figure S4, Supporting Information). First, the user would need experimental data for a calibration curve, obtaining both AFM heights and optical micrographs of the samples of interest at their minimum and maximum depth extrema (Figure S4B, Supporting Information). In our situation, we obtained upward and downward deflected AFM data of the bulges at the beginning and an intermediary step of the experiment. We show the trends for two different bulges on the substrate, exemplifying the need to have a different calibration curve for each bulge. Of importance, the user needs to ensure that the lighting conditions are consistent throughout the course of the experiment.

Then, the user would create a calibration curve between AFM and RGB values (Figure S4C, Supporting Information). This involves first correlating the AFM data with the RGB values along the diameter of a single bulge, ensuring that their indices are aligned and that only data corresponding to the bulge is preserved. If using MATLAB, the Curve Fitting Toolbox is suitable for creating the Fourier series fits, and the experimental data corresponding to the minimum and maximum depth extrema should be used.

Next, to determine the algorithmic parameters, the user could use the algorithm for a 2D slice of the bulge and input a single diameter's RGB data with a known AFM result. They can then tune parameters related in proceeding from Figure 3C‐ii,iii so that the result of the algorithm aligns with the known AFM result (Figure S4D, Supporting Information). These parameters correspond to the ratio of absolute number of data points and the R2 of fits to a positive and negative parabola. Depending on a user‐chosen threshold, the algorithm determines whether to proceed with the positive or negative parabola. When determining the appropriate parameters, we used parity of the calculation with the known AFM data points as the metric.

Finally, the algorithm is ready to process the optical micrographs taken throughout the experiment. The user can take the DWM mean approach, first obtaining a result for the 3, 4, and 5‐term fits by changing the input calibration curve and then calculating the DWM (Figure S4F, Supporting Information). We find that, in order to maximize the accuracy of the calculations, it is best to process video frames/images taken within quick succession of each other, thus enabling the user to use physical intuition to evaluate the results.

In our experiment, our analyses show that the bulges, during helium flow, remained upward deflected, but over time became downward deflected. The final downward deflected states are similar to their deflection before nitrogen inflation, all of them ranging from 63% to 83% that of the original depth. Thus, this analysis helps to elucidate that the deflation does not occur immediately, and that it provides us with the hypothesis that the helium flow could have un‐adhered the films from the substrates, leading to the deflation.

We now discuss how to apply our algorithm to systems not exemplified by the reference He experiment. When using materials with other refractive indices and thicknesses, one needs to consider what the intensity of reflected visible light is for different well geometries. As the detector is an optical microscope camera, we are most concerned with visible wavelengths. One can use Equations (1), (2), (3), (4), (5), (6), (7), (8) to calculate what the intensity of reflected visible light is for various well depths and then calculate how the RGB values would change with well depth as described within the same section. We have demonstrated this with graphene in Figure S5 (Supporting Information). If the well depth is not easily manipulated to make sure that the reflected light intensity yields discernible RGB trends, then one could consider introducing additional reflective layers; however, this would then potentially have an impact on the experimental interpretation.

Additionally, we consider how different environmental conditions could affect the experimental considerations. The environmental chamber for the samples requires a transparent viewing window for the microscope camera to observe the bulges. The window thickness and environmental conditions could affect the visibility of the bulge, such as by blurring the image, as well as having varying distances between the window and sample (Figure S5, Supporting Information) how different glass covers affect the image. This is compensated for by using a calibration curve for each individual bulge, and has a notable effect on the calibration curves used in Figure 5 (Figure S3, Supporting Information). Moreover, if changing the gas environment, say to a chemically corrosive gas, one needs to ensure that the conditions cannot result in condensation on the viewing window. There also needs to be consideration for the new refractive index of the surrounding gas as well as the chamber's ability to contain the gas.

## 4 Conclusion

In this study, we derive and validate a set of semi‐empirical approximations using the Fresnel equations applicable to thin film systems and develop an algorithm to convert optical micrographs into thin film depth information. We apply our algorithm to a gas permeability bulge test experiment. Instead of Hencky's solution, we approximate the shape of the bulges using parabolic deflections. The results of our algorithm enable us to greatly improve our understanding of the filling and sealing behavior of bulge test systems and extend this technique to non‐atmospheric pressures and temperatures.

## 5 Experimental Section

### 2DPA‐1 Synthesis

To synthesize 2DPA‐1 powder, 1 mmol of trimesoyl chloride was combined with 1 mmol of melamine in a 40‐mL glass vial. Then, a magnetic stir bar was used, inside an N2 environment, and stirred the contents of the vial with 9 mL of n‐methyl‐pyrrolidinone and 1 mL of pyridine. The reaction was sealed with a solvent resistant cap and allowed it to stir at room temperature for 12 h between 350 and 500 rpm. Afterward, 20 mL of isopropanol was added to quench the reaction and stir for 30 more min. Then, the vial was removed from the glovebox, filter the solution with a Buchner funnel, and washed repeatedly with isopropanol. The rinsed residue inside the funnel was then collected and stirred with 10 mL of deionized water and 10 mL of acetone inside a 20 mL glass vial for 8 h at room temperature. The resulting mixture was again filtered and washed multiple times with acetone. Finally, the resulting solid was collected, dried in a vacuum oven at 100 °C for 3 h, and ground into a powder.

### Thin Film Preparation for Bulge Tests

The base substrate of the thin films, silicon/silicon oxide (Si/SiO_2_) wafers, were cleaned with a 5‐min sonication bath in acetone, a subsequent 5‐min sonication batch in isopropanol, and dried with N_2_. Then, a layer of 10 wt.% polystyrene in anisole was spin‐coated onto the substrate at 2000 rpm for 1 min. Next, the system was annealed on a hot plate at 110 °C for 15 min under a glass petri dish, which minimizes the collection of debris on the PS layer. Then, with the PS‐coated substrate, 2DPA‐1 dissolved in TFA was spin‐coated at the same spin settings, controlling the film thickness between 4 and 60 nm by adjusting the solution concentration. Finally, the 2DPA‐1 and PS‐coated substrate was annealed on a hot plate at 50 °C for 5 min.

After preparing the PS‐supported 2DPA‐1 thin films, the films were wet‐transferred them onto Si/SiO_2_ etched substrates for bulge testing. First, the etched substrates were cleaned with the same procedure for the base substrates. Then, using a razor blade, an area of 2DPA‐1/PS film was isolated from the rest of the film. An edge of the isolated film was exposed to water, which gets in‐between the PS layer and the base substrate. Next, the isolated film was suspended onto the surface of water, where the PS layer contacts the water, and contact the etched substrate with the 2DPA‐1 layer of the isolated film, quickly scooping the isolated film out of the water onto the etched substrate. The film‐covered substrate was then dried overnight on a hot plate at 50 °C. The drying temperature affects the deflection of the bulges on the sample, where 50 °C results in downward‐deflected bulges.

Finally, the PS layer was removed from the 2DPA‐1/etched substrate. After drying, the sample was placed in a glass petri dish with a 20 mL mixture of 25% v/v chloroform in hexane for 4 h. Then, the sample was transferred into a 20‐mL mixture of 30% v/v chloroform in hexane for another 4 h. The resulting samples were then gently rinsed in hexane, dried, and then stored at ambient conditions.

### Atomic Force Microscopy

To measure the deflections of bulge test samples, a Cypher S in tapping mode was used with an AC240 tip. A setpoint of 800 mV, scan rate of 1.5 Hz, a scan area of 20 µm, and a resolution of 256 × 256 points and lines were used. Each bulge on the substrates is 8.5 µm in diameter.

### Environmental Chamber

A commercial Linkam THMS600 environmental stage was used for all experiments.

## Conflict of Interest

The authors declare no conflict of interest.

## Supporting information



Supporting Information

## Data Availability

The data that support the findings of this study are available from the corresponding author upon reasonable request.
