# Supplementary material for: Interferometric Deflection Analysis of Suspended 2D Polyaramid Thin Films
Source: Small Methods. 2025 Dec 5;10(1):e01543. doi: 10.1002/smtd.202501543 (PMC12790372; doi:10.1002/smtd.202501543)
Supplement: Supplementary file 1 — Supporting Information [file SMTD-10-e01543-s001.pdf]

Supplementary Information for

Interferometric Measurements of Two Dimensional Polyaramid Thin Films

Michelle Quien *et al.*

Corresponding Author: Michael S. Strano, strano@mit.edu

The PDF file includes:

Supplementary Text

Figs. S1 to S5

Supplementary References

Refractive Indices of 2DPA-1

We measured the refractive index of a 15-nm, 35-nm, and 65-nm thick spin-coated film of 2DPA-1 using a Filmetrics F20 reflectometer (*Figure S1 A*). The data was measured from 380 to 780 nm. The experimental refractive index values for 35-nm were used in calculating *Figure 2B* of the main text, where n, the complex refractive index, has real component n_r and imaginary component k.

We additionally sought to calculate how the variation in a spin-coated film would affect the refractive index. We empirically fit n_r and k with two parameters, the thickness and the wavelength (*Figure S1 A*). We used Matlab's Curve Fitting Toolbox to fit a polynomial surface; the resultant fits and parameters are shown below:

$$n_r(d,\lambda) = p_{00} + p_{10}\,\lambda + p_{01}\,d + p_{20}\,\lambda^2 + p_{11}\,\lambda\,d + p_{02}\,d^2 + p_{30}\,\lambda^3 + p_{21}\,\lambda^2\,d + p_{12}\,\lambda\,d^2 \quad \text{(S1)}$$

$$k(d,\lambda) = p_{00} + p_{10}\,\lambda + p_{01}\,d + p_{20}\,\lambda^2 + p_{11}\,\lambda\,d + p_{02}\,d^2 + p_{30}\,\lambda^3 + p_{21}\,\lambda^2\,d + p_{12}\,\lambda\,d^2 + p_{40}\,\lambda^4 + p_{31}\,\lambda^3\,d + p_{22}\,\lambda^2\,d^2 \quad \text{(S2)}$$

$n_r(d,\lambda)$ parameters		$k(d,\lambda)$ parameters	
p_{00}	1.5899	p_{00}	0.0004
p_{10}	0.0049	p_{10}	-0.0014
p_{01}	0.112	p_{01}	-0.0008
p_{20}	0.0013	p_{20}	0.0012
p_{11}	-0.0039	p_{11}	0.0008
p_{02}	-0.0541	p_{02}	0.0004
p_{30}	-0.001	p_{30}	-0.0005
p_{21}	0.0025	p_{21}	0.0007
p_{12}	-0.0026	p_{12}	0.0009
		p_{40}	0.0002
		p_{31}	-0.0004
		p_{22}	-0.0007

Table S1: Polynomial Surface Fitting Parameters for Refractive Indices

We also have previously reported the surface roughness of 2DPA-1 spin-coated films to be 543pm. Using *Equations S1 and S2,* we can then calculate the impact of this roughness on the refractive index components, and find that it affects n_r to the 3rd decimal point and has no notable effect on k (*Figure S1 C*).

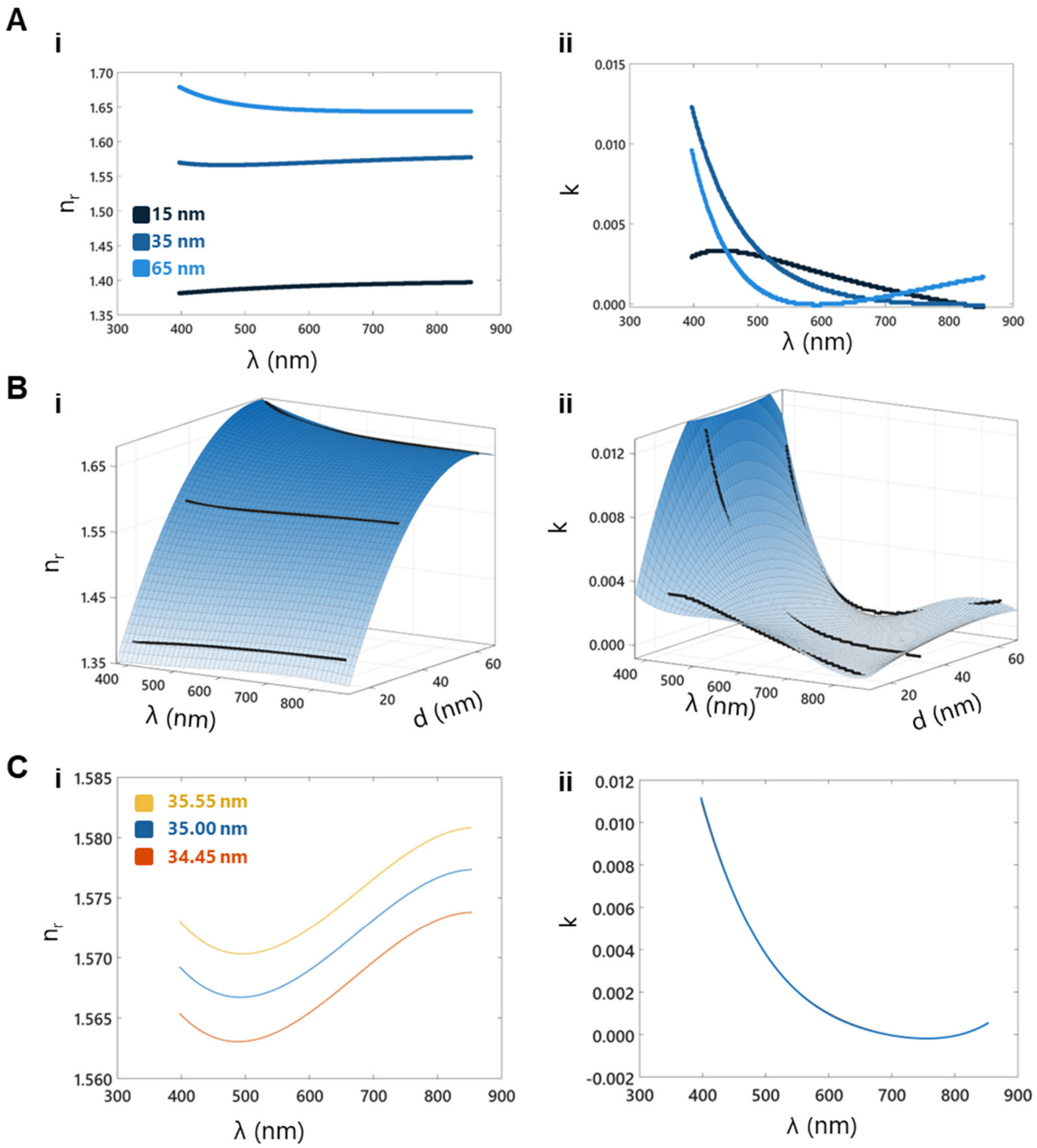

Figure S1.

(A) Real (i) and imaginary (ii) components of the refractive index of a 15-nm, 35-nm, and 65-nm thick 2DPA-1 spin-coated film from 380 to 780 nm

(B) Empirically fit relationship of the real (i) and imaginary (ii) components with the wavelength and film thickness

(C) Variation in the real (i) and imaginary (ii) components of a 35-nm thick film using experimental surface roughness.

Theory for converting Light Interferometry into RGB values

To convert reflected light intensities into RGB values, as in Fig 2B, we used theory published by the International Commission on Illumination (CIE) in 1931 for the relationship between the visible spectrum and the CIE RGB. First, the reflected light intensity is used to calculate the CIE XYZ color space:

$$X = \frac{K}{N} \int_{\lambda=380nm}^{\lambda=780nm} S(\lambda)\, I(\lambda)\, \bar{x}(\lambda)\, d\lambda \tag{S3}$$

$$Y = \frac{K}{N} \int_{\lambda=380nm}^{\lambda=780nm} S(\lambda)\, I(\lambda)\, \bar{y}(\lambda)\, d\lambda \tag{S4}$$

$$Z = \frac{K}{N} \int_{\lambda=380nm}^{\lambda=780nm} S(\lambda)\, I(\lambda)\, \bar{z}(\lambda)\, d\lambda \tag{S5}$$

where

$$N = \int_{\lambda=380nm}^{\lambda=780nm} I(\lambda)\, \bar{y}(\lambda)\, d\lambda \tag{S6}$$

where K is a scaling factor, set to 1 in this case, λ is the wavelength of light in the visible range, S is the spectral reflectance of the object being measured, I is the spectral power distribution of the illuminant, and $\bar{x}(\lambda)$, $\bar{y}(\lambda)$, and $\bar{z}(\lambda)$ are color matching functions. We used the "Spectral and XYZ Color Functions" toolbox[1] within MATLAB to obtain the color matching functions. The spectral power distribution of the illuminant is the intensity of light output at each wavelength of the light source. In this case, our light source is a tungsten halogen bulb, and we used previously reported experimental values.[2,3] After computing X, Y, and Z, we used the "xyz2rgb" function from Matlab's Image Processing Toolbox to convert CIE XYZ into CIE RGB.

Expansion & Approximations to Hencky's Solution

Herein is the rationale for fitting our data set to a second-order polynomial instead of the full Hencky's solution and how it would be applicable for materials with positive Poisson's ratios. Hencky's solution is written as an infinite summation, and can be expanded as:

$$\frac{\delta}{d/2}(\mathrm{r}) = \left(\frac{\Delta p}{2E}\right)^{\frac{1}{3}} \left[a_0\left(1-\left(\frac{r}{d/2}\right)^2\right) + a_2\left(1-\left(\frac{r}{d/2}\right)^4\right) + a_4\left(1-\left(\frac{r}{d/2}\right)^6\right) + a_6\left(1-\left(\frac{r}{d/2}\right)^8\right) + \cdots\right] \tag{S7}$$

The prefactor terms, generalized as a_{2n}, wherein a_0 corresponds to the second-order polynomial term, a_2 corresponds to the fourth-order term, etc., are related to the Poisson's ratio, ν, via boundary conditions. When imposing the condition that the bulge is clamped to the edge of the well, it is shown that:[4]

$$
\left.\begin{aligned}
a_0 &= \frac{1}{b_0} \\
a_2 &= \frac{1}{2\, b_0^4} \\
a_4 &= \frac{5}{9\, b_0^7} \\
a_6 &= \frac{55}{72\, b_0^{10}} \\
a_8 &= \frac{7}{6\, b_0^{13}} \\
a_{10} &= \frac{205}{108\, b_0^{13}} \\
&\vdots
\end{aligned}\right\}
\tag{S8}
$$

where

$$
(1-\nu)b_0 - \frac{(3-\nu)}{b_0^2} - \frac{2(5-\nu)}{3\, b_0^5} - \frac{13(7-\nu)}{18\, b_0^8} - \frac{17(9-\nu)}{18\, b_0^{11}} - \frac{37(11-\nu)}{27\, b_0^{14}} - \frac{1205(13-\nu)}{567\, b_0^{17}} - \frac{219241(15-\nu)}{63504\, b_0^{20}} - \frac{6634069(17-\nu)}{1143072\, b_0^{23}} - \frac{51523763(19-\nu)}{5143824\, b_0^{26}} - \frac{998796305(21-\nu)}{56582064\, b_0^{29}} + \cdots = 0 \tag{S9}
$$

For a given ν, there is a singular value of b_0 for which $b_0 > 1$.[4] In solving the first 11 terms of *Equation S9*, we obtain the range of b_0 for $\nu \in [0, 0.5]$ (*Figure S2 A i*), the ranges of a_0, a_2, a_4, and a_6 (*Figure S2 A ii*), and the ratios of a_0/a_2 and a_0/a_4 (*Figure S2 A iii*). We show that the prefactor for the second-order term is thus at least 8.5 times that of the fourth-order and sixth-order terms, giving rationale for why the second-order term is the most dominant term within Hencky's solution .

We also compared the shape and area of the curve of Hencky's solution if only the a_0 term is maintained (1-term approximation), only the a_0 and a_2 terms are maintained (2-term approximation), only the a_0, a_2, and a_4 terms are maintained (3-term approximation), and only if the a_0, a_2, a_4, and a_6 terms are maintained (4-term approximation) for $\nu = 0$ (*Figure S2 B i*) and for $\nu = 0.5$ (*Figure S2 B ii*). We quantified the difference in the approximations by calculating the percent difference in area for the 1-term, 2-term, and 3-term approximations with the 4-term approximation (*Figure S2 C*) and find them to deviate by less than 3% for $\nu \in [0, 0.5]$. We also quantified the difference in δ values, $\Delta\delta$, between the 1-term and 4-term approximations, 2-term and 4-term approximations, and 3-term and 4-term approximations (*Figure S2 D i-iii*). We normalized the max height of δ to be 1 at $r = 0$ in all cases and find the maximum difference to be around 0.035 (*Figure S2 D i*), or 3.5% that of the maximum height.

Thus, the usage of only the 1-term approximation to Hencky's solution leads to low error in terms of the area under the curve of the bulges as well as the deflection along the curve.

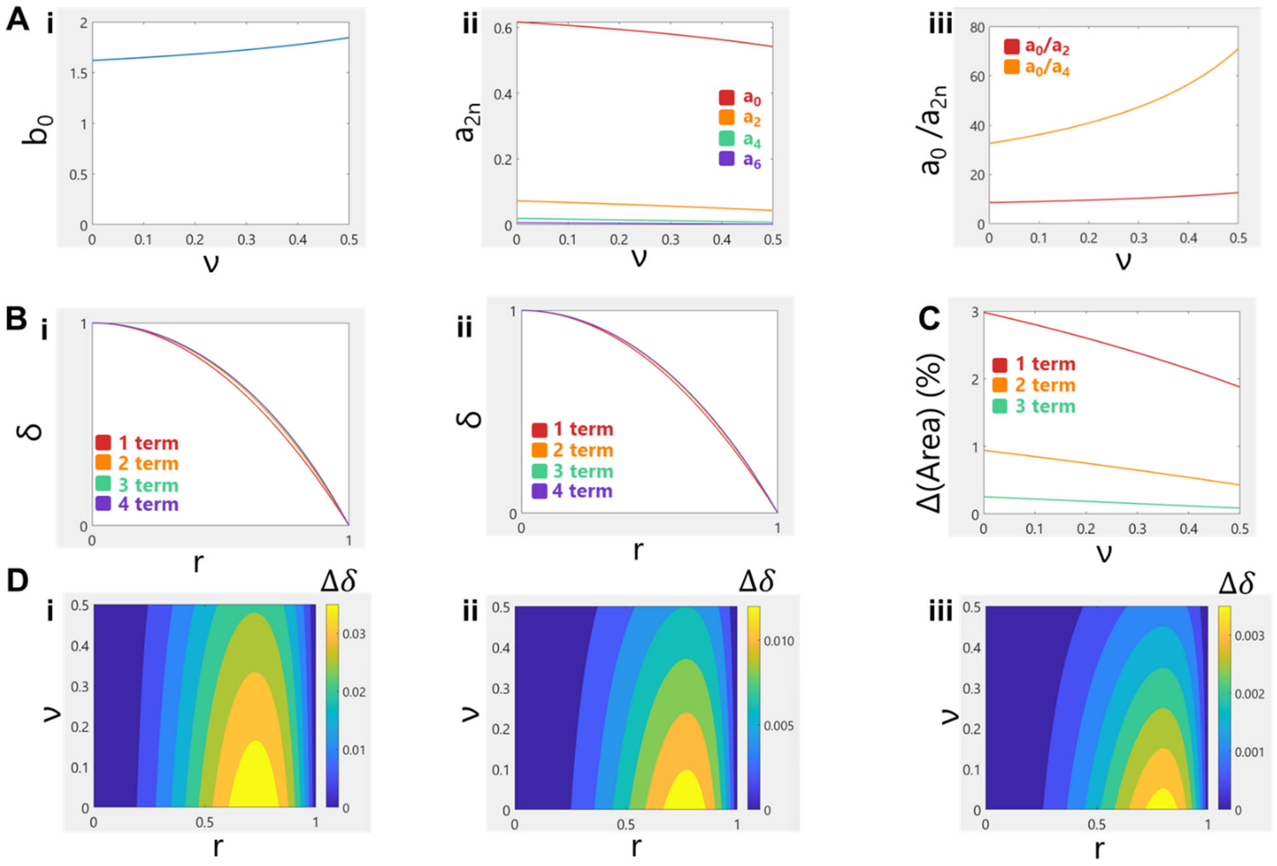

Figure S2.

A) Calculations of b_0 **(i)**, a_{2n} for $n \in [0,3]$ **(ii)** and a_0/a_{2n} for $n \in [1,2]$ **(iii)** for the range $\nu \in [0.0,0.5]$ using the first 11 terms of Equation S7.

B) Calculations of dimensionless $\delta(r)$, where $\delta, r \in [0,1]$, for $\nu = 0.0$ **(i)** and $\nu = 0.5$ **(ii)**, using the 1-term (red), 2-term (orange), 3-term (green), and 4-term (purple) approximations.

C) Percent difference in total area under the $\delta(r)$ curve, Δ(Area), for $\nu \in [0.0, 0.5]$ between the 1-term and 4-term (red), 2-term and 4-term (orange), and 3-term and 4-term (green) approximations.

D) Contour plots of the absolute difference in the $\delta(r)$ curve of the 1-term and 4-term **(i)**, 2-term and 4-term **(ii)**, and 3-term and 4-term **(iii)** approximations along the profile of the bulge ($r \in [0,1]$) and for $\nu \in [0.0,0.5]$.

RGB curves for all 8 bulges

In *Figure S3*, we show the individual R, G, and B vs deflection correlations for each analyzed bulge, with the 3, 4, 5, and 6-term Fourier fits for each. The gray region is the interpolated region which has no experimental data points and is most likely to differ between each of the fits. We also show the parity of each of the Fourier fits. We found that the 6-term fit is the farthest from parity, attributed to over-fitting of data, and thus do not include it in the DWM analysis in the main text.

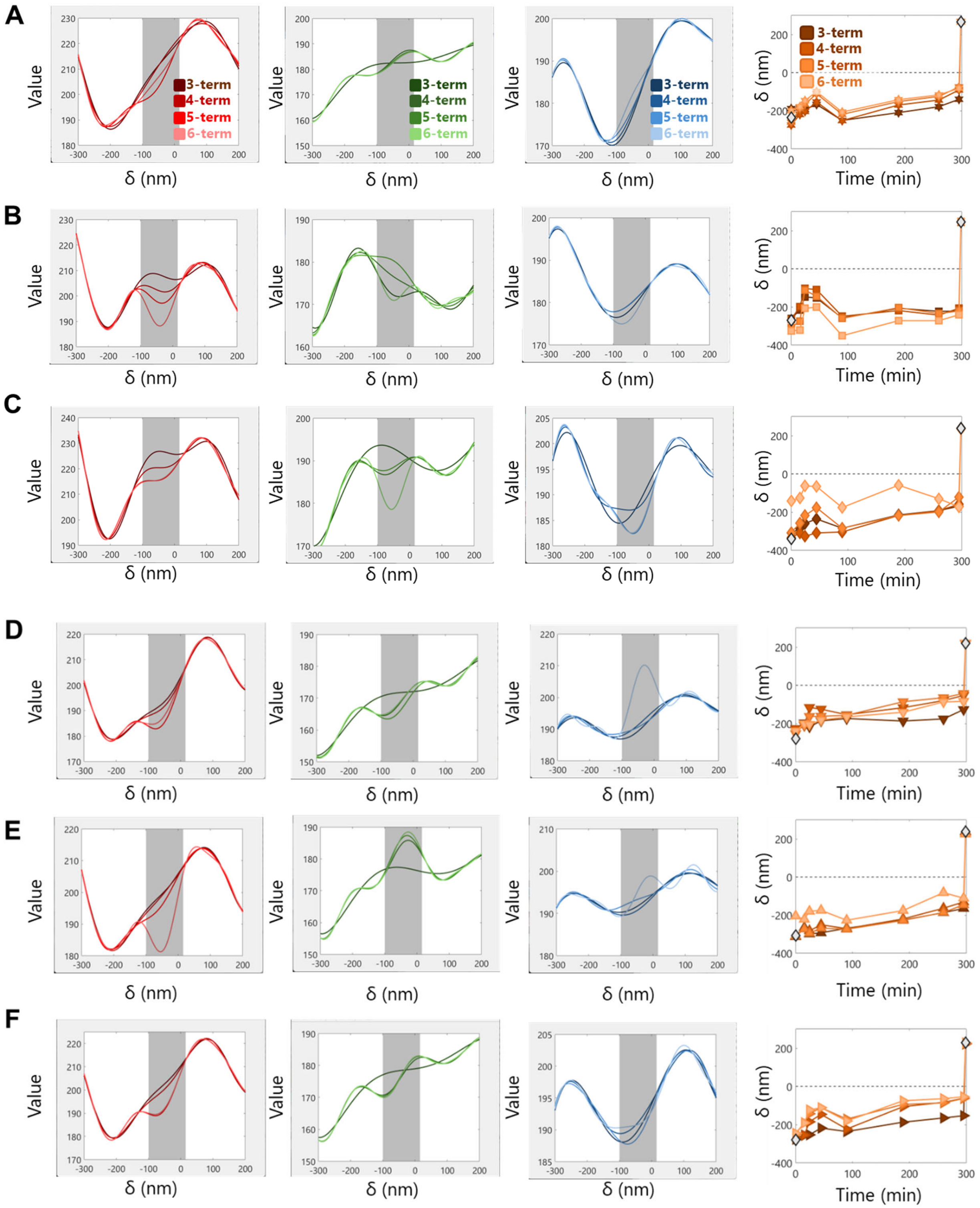
A
B
C
D
E
F
Value
δ (nm)
3-term
4-term
5-term
6-term
Time (min)

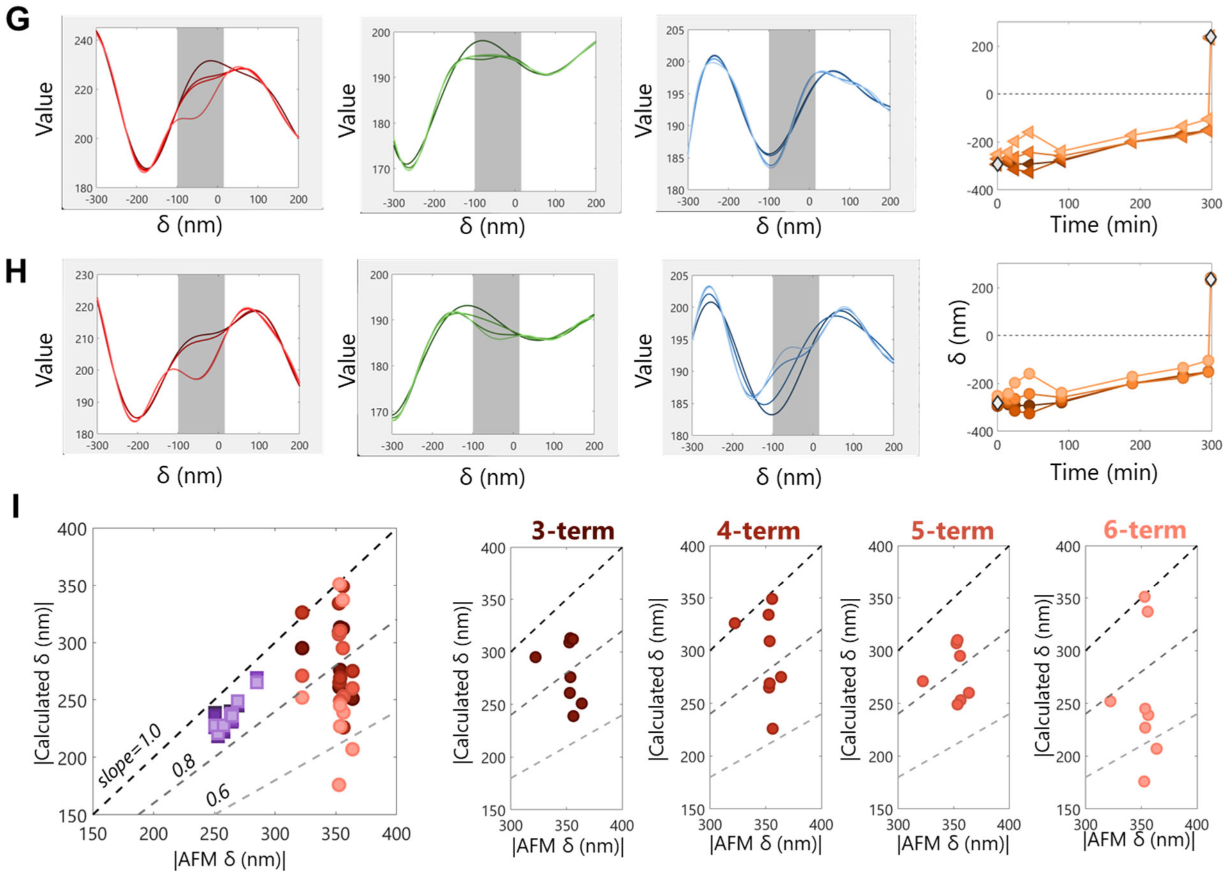

Figure S3. Semi-Empirical Fittings of 8 Bulges

A-H) 3, 4, 5, and 6-term Fourier series fittings of the R, G, and B channels for each individual bulge as well as the resulting deflection extrema for all 9 optical micrographs. The gray shaded regions indicate where there is a lack of experimental data points.

I) Parity plot of the absolute value of the initial and final data points for all 8 studied bulges. Dashed lines have slopes 1.0, 0.8, and 0.6, darkest to lightest, respectively. Purple data sets correspond to the positive extrema while red data sets correspond to negative extrema. Four sets of each color are shown for the 3-term, 4-term, 5-term, and 6-term Fourier fits (darkest to lightest, respectively). (Right) Negative extrema results for each Fourier fit, isolated from the main parity plot.

Applying the Algorithm: Tracking Bulge Dynamics

Herein, we detail an example for how to use the MATLAB algorithm for converting optical micrographs into height information. We discuss this in the context of an experiment on 2DPA-1 bulge test samples with helium (He) gas. We monitored 8 bulges on a bulge test substrate (*Figure S4 A*) which were initially pressurized with 300 kPa gauge pressure of nitrogen for 300 minutes and then, 24 hours later, subjected to a light He flow for 20 minutes that had an undetectable pressure on our standard pressure gauge.

For each bulge of interest, we obtained both the RGB and height values of an upwards and downwards deflected state (*Figure S4 B*). We show the trends for two different bulges on the substrate, exemplifying the need to have a different calibration curve for each bulge. We extracted the RGB values from the background-subtracted images of each bulge and used indexing within MATLAB to match the lengths of the height and RGB arrays, reducing the resolution on the larger array to match that of the shorter array.

We then created a calibration curve for each bulge of interest and made a 3-term, 4-term, and 5-term Fourier fit to each (*Figure S4 C*). This involved concatenating both the RGB and height data for upwards and downwards deflected states into a single array. This was then input into MATLAB's Curve Fitting Toolbox, from which we output the Fourier fits as *cfit* data types. At this point in the process, we saved all of our variables within a .mat file.

We then determined the appropriate algorithmic parameters for each specific bulge by using images of the bulge wherein we knew the corresponding AFM height and inputting 2D slices into the algorithm. We used parity as a metric to determine when the algorithmic parameters resulted in the closest fit with the AFM data, where we desire parity close to 1 (*Figure S4 D*). We found that for a given bulge, there was a range of metrics that resulted in parity closest to 1, and thus would use the information for both the downwards and upwards deflected data points to narrow the algorithmic parameters.

Finally, once we determined the algorithmic parameters, we processed video frames and images for each bulge throughout the course of our experiment and used a parity plot to quantify the fitting (*Figure S4 E & F*). We include an analysis of the nitrogen pressurization for the parity analysis and record images of the bulges for around 40 minutes after He flow. Using AFM, we can verify that all 8 bulges became positively deflected after nitrogen pressurization, and after He flow, all 8 bulges became negatively deflected within 19 hours (1140 minutes). The analysis implies that there could be bulge deflation over the course of the He flow (*Figure S4 F ii, iii, v, vii*); however, given that the deflection analyzed from the surrounding video frames is consistent, we would attribute this apparent deflation as a result of video blurring or noise, and thus discount it. Our analysis enables us to note that, during the He flow and within 40 minutes afterwards, all 8 bulges stayed positively deflected. Thus, this offers insight into the effect of the He flow; the final negative deflection is not caused immediately by the flow and is more likely to be caused by a slower mechanism.

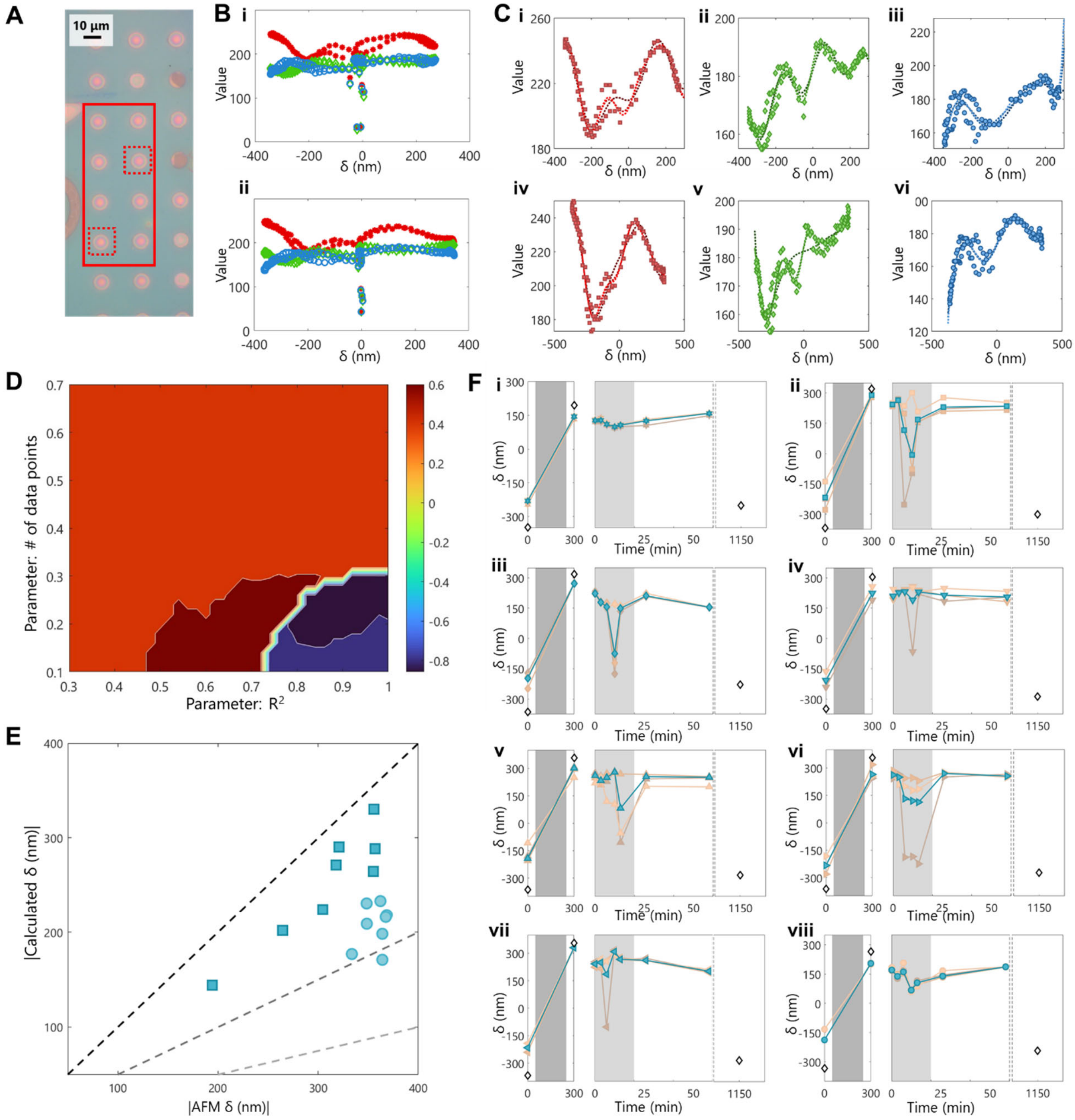

Figure S4. Application of Hue Analysis Algorithm to He Experiment

A) Optical micrograph of the bulge test sample used for the He flow experiment. The red solid box encompasses all 8 monitored bulges, while the red dotted box encompasses the 2 bulges used in Figure S4 B and C.

B) Correlation between RGB pixel values and the corresponding AFM measured deflection for both the upward and downward deflected states of 2 separate bulges. Red asterisks correspond to

the R component of RGB, green diamonds correspond to G, and blue open circles correspond to B.

C) Comparison of fit Fourier sum equations (lines) with experimentally measured RGB pixel values (markers) for two separate bulges. Shown for each RGB channel (left, R; center, G; right, B). Each dashed line represents a different Fourier fit (3-term, 4-term, and 5-term, darkest to lightest respectively)

D) Contour plot showing the impact of two algorithmic threshold parameters, one related to relative R^2 values and the other related to relative number of data points, on the parity of the algorithmically calculated deflection with the experimental AFM result. Corresponds to the downward deflected state of a bulge.

E) Parity plot of the absolute value of the positively and negatively deflected data points for all 8 studied bulges, for the DWM of the 3-term, 4-term, and 5-term Fourier fits. Dashed lines have slopes 1.0, 0.5, and 0.25, darkest to lightest, respectively. Square data sets correspond to the positive extrema while circle data sets correspond to negative extrema.

F) Calculated DWM of the deflection extrema (blue markers) are plotted for one full pressurization experiment on all 8 studied bulges. Within each plot, we include the 3-term, 4-term, and 5-term Fourier fits, at high transparency (darkest to lightest, respectively). A black dotted line delineates positive from negative height values. Each marker shape corresponds to one bulge. The extrema of measured AFM profiles (white diamonds) are shown where obtained. The dark gray shading indicates when the bulges were pressurized by nitrogen while the light gray shading indicates when the bulges were subjected to He flow.

Applying the Algorithm to Other Systems

In *Figure S5*, we show some considerations that should be accounted for to extend this technique beyond our 2DPA-1 bulge test platform. First, in using a film with an alternate thickness/material, one can calculate beforehand using *Equations 1-8* how this will impact the observed RGB values. We show in *Figure S5 A* how using a single layer of graphene (d=0.4nm, n=2.6-1.3i)[5] affects the reflected intensity of visible light and then the captured RGB pixel values. Then, we show the affect of different glass thicknesses in *Figure S5 B* on the captured images, wherein increasing thickness increases the bluriness of the image and changes the reflected light intensity.

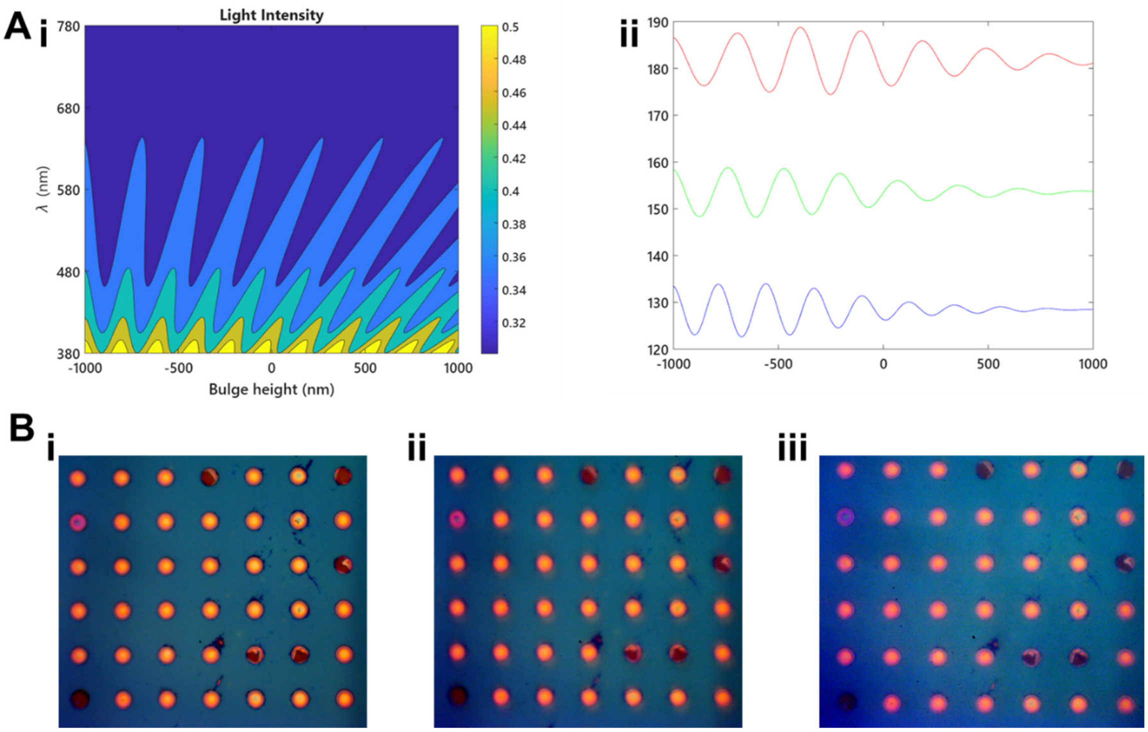

Figure S5. Considerations for Other Systems

A) Correlation between light intensity (i) and RGB values (ii) with bulge depth for a single-layer graphene film

B) Optical images of 2DPA-1 films with (i) no cover glass, (ii) a 300um thick glass, and (iii) a 500um thick glass

Supplementary References

(1) Mather, J. *Spectral and XYZ Color Functions*. https://www.mathworks.com/matlabcentral/fileexchange/7021-spectral-and-xyz-color-functions (accessed 2025-04-21).
(2) *ZEISS Microscopy Online Campus | Tungsten-Halogen Lamps*. https://zeiss-campus.magnet.fsu.edu/articles/lightsources/tungstenhalogen.html (accessed 2025-04-21).
(3) *Tungsten-Halogen Light Sources*. https://www.thorlabs.com (accessed 2025-04-21).
(4) Fichter, W. B. Some Solutions for the Large Deflections of Uniformly Loaded Circular Membranes.
(5) Blake, P.; Hill, E. W.; Castro Neto, A. H.; Novoselov, K. S.; Jiang, D.; Yang, R.; Booth, T. J.; Geim, A. K. Making Graphene Visible. *Appl. Phys. Lett.* **2007**, *91* (6), 063124. https://doi.org/10.1063/1.2768624.
